# Zoledronic Acid Enhanced the Antitumor Effect of Cisplatin on Orthotopic Osteosarcoma by ROS-PI3K/AKT Signaling and Attenuated Osteolysis

**DOI:** 10.1155/2021/6661534

**Published:** 2021-03-30

**Authors:** Liang Liu, Huan Geng, Chengjie Mei, Liaobin Chen

**Affiliations:** ^1^Department of Orthopedic Surgery, Zhongnan Hospital of Wuhan University, Wuhan 430071, China; ^2^Department of Hepatobiliary and Pancreatic Surgery, Zhongnan Hospital of Wuhan University, Wuhan 430071, China

## Abstract

Osteoclasts can interact with osteosarcoma to promote the growth of osteosarcoma. Cisplatin is common in adjuvant chemotherapy of osteosarcoma. However, due to chemoresistance, the efficacy is profoundly limited. Previous studies have found that zoledronic acid (ZA) has osteoclast activation inhibition and antitumor effect. However, the combined effect of ZA and cisplatin on osteosarcoma remains unclear. In vitro, the effects of ZA and cisplatin alone or in combination on 143B cell activity, proliferation, apoptosis, and ROS-PI3K/AKT signaling were detected. At the same time, the effect of ZA and cisplatin on osteoclast formation, survival, and activity was detected by TRAP staining and bone plate absorption test. These were further verified in mice. The results showed that in vitro, compared with the single treatment and control, the combination of ZA and cisplatin could significantly inhibit the activity and proliferation of 143B cells and induced their apoptosis and further promoted the generation of ROS and inhibited the phosphorylation of PI3K and AKT. ROS scavenger and the agonist of the PI3K/AKT pathway could reverse these results. In addition, cisplatin in synergy with ZA could significantly inhibit osteoclast formation and survival to reduce bone plate absorption. In vivo, compared with the single group, the tumor volume and cell proliferation were significantly reduced, apoptosis and necrosis of tumor cells increased, and TRAP^+^ osteoclasts and osteolysis destruction decreased in the combined group. In conclusion, ZA enhanced the antitumor effect of cisplatin on osteosarcoma by ROS-PI3K/AKT signaling, reducing the chemoresistance and osteoclast activation to enhance chemotherapy and inhibit osteolysis. And this present study raised the possibility that combining ZA and cisplatin may represent a novel strategy against osteosarcoma.

## 1. Introduction

Osteosarcoma, common in children and adolescents, is a primary malignant tumor, most of which occur in the metaphysis of long bones [1]. Clinically, surgical excision, adjuvant chemotherapy, and radiotherapy are the most important methods for the treatment of osteosarcoma [2]. With the development of surgical techniques and the use of adjuvant chemotherapy, the survival rate of patients with osteosarcoma has been greatly improved [3]. However, 5-year survival rates were reported to be still only 15 to 30% for patients with chemotherapy resistance or metastasis [3].

Cisplatin is a classical chemotherapy drug used to treat osteosarcoma [4]. In mechanism, on the one hand, cisplatin causes single or double strand damage to DNA by binding to bases on DNA molecules, which affects DNA replication to inhibit tumor cell proliferation. On the other hand, cisplatin plays an antitumor role through cell apoptosis mediated by organelles such as mitochondria and endoplasmic reticulum [5, 6]. But due to drug resistance and side effects, the efficacy of cisplatin in osteosarcoma has been deeply limited [5]. At present, the common multidrug combined chemotherapy can partially improve the effect on killing osteosarcoma cells, such as cisplatin, methotrexate (MTX), Adriamycin (ADM), and ifosfamide (IFO) [7]. However, there is no combined strategy for osteosarcoma that not only can significantly enhance the efficacy but also inhibit these side effects of the combined drugs. Therefore, a novel and more effective treatment with lower side effects is needed to complement the current strategy to improve overall survival.

Osteosarcoma cells derive from osteoblasts. However, it has been found that osteoclasts also play an important role in the occurrence and development of osteosarcoma [8]. Osteoclasts and their progenitors express RANK receptors, while osteosarcoma cells can secrete a large number of RANK ligands (RANKLs) [8]. RANKLs promote the precursors to differentiate into osteoclasts and activate them, which then leads to osteolysis and releases a variety of growth factors, such as transforming growth factor beta (TGF*β*), insulin-like growth factors (IGFs), and fibroblast growth factors (FGFs) [9–11]. In turn, these factors promote the growth of tumor cells to further promote the formation and activation of osteoclasts, thus forming a “vicious circle” [11]. Zoledronic acid (ZA), the third-generation clinical applied bisphosphonate with strong bone affinity, is widely used in the treatment of metabolic and metastatic bone diseases nowadays [12]. In mechanism, ZA can dwindle osteoclast function or enhance osteoclast apoptosis [13, 14]. Moreover, previous studies have found that ZA is the most potential bone resorption inhibitor for bone metastases [15]. Preclinical data also found that ZA can inhibit angiogenesis, tumor cell adhesion, and invasion of extracellular matrix to inhibit a variety of tumors, such as osteosarcoma, cervical cancer, and breast cancer [7, 16, 17]. However, as a classic antiosteoporosis drug, whether ZA combining cisplatin can enhance the inhibition of osteosarcoma growth remains unclear.

In this study, we examined the efficacy of combined therapy with cisplatin and ZA. We demonstrated that combination with cisplatin and ZA enhanced the chemotherapy effect of cisplatin on osteosarcoma. It could further induce the apoptosis and proliferation inhibition of osteosarcoma cells caused by cisplatin, and the mechanism of synergistic effect enhancement was mainly related to oxidative stress and PI3K/AKT inactivation, which play a crucial role in cell survival and proliferation. Furthermore, we confirmed the synergistic antitumor effect of combined treatment with ZA and cisplatin in mice, and it inhibited osteoclasts to break the “vicious circle” between osteoclasts and osteosarcoma cells and reduce osteolysis (an active resorption of bone matrix by osteoclasts).

## 2. Material and Methods

### 2.1. Reagents

Zoledronic acid (CAS No. 118072-93-8), cisplatin (CAS No. 15663-27-1), and 740 Y-P (CAS No. 1236188-16-1) were obtained from MedChemExpress (Wuhan, China). Isoflurane was obtained from Baxter Healthcare Co. (Deerfield, IL, USA). TRAP staining kit (HR0561) was purchased from Biolab Technology Co., Ltd. (Beijing, China). Reactive oxygen species (ROS) test kit (DHE) (HR8821) was obtained from Biolab (Beijing, China). Phospho-PI3 kinase (p-PI3K) (4228S) and AKT (4691S) antibodies were obtained from Cell Signaling Technology Co., Ltd. Phospho-AKT (AP0637), MRP-1 (A1703), MDR1 (A19093), and PI3K (A0265) antibodies were obtained from ABclonal Co., Ltd. (Wuhan, China). Goat polyclonal secondary antibody to rabbit IgG-H&L (Alexa Fluor® 488) (ab150077) was obtained from Abcam. Annexin V-FITC/PI kit (BB-4101-50T) was purchased from BestBio (Shanghai, China); Ki-67 (GB13030-2), TMR (red) Tunel Cell Apoptosis Detection Kit (G1502), crystal violet (G1014), and CCK-8 kit (G4103) were purchased from Servicebio Co., Ltd. (Wuhan, China). TRIzol (CSA No. 5346994) was obtained from Invitrogen Co. (Carlsbad, CA, United States). Reverse transcription and real-time quantitative polymerase chain reaction (RT-qPCR) kits were obtained from TaKaRa Biotechnology (Dalian, China). The SYBR Green dye was purchased from Applied Biosystems by Thermo Fisher Scientific (ABI) (Foster City, CA, USA). The other chemicals and agents were analytical grade.

### 2.2. Cell Culture and Treatment

Human osteosarcoma 143B cell lines were purchased from Mingzhou Biological Technology Co., Ltd. (Zhejiang, China). Cells resistant to cisplatin were established by exposing the drug-sensitive 143B cells to stepwise increasing concentrations of cisplatin. All cells were cultured at 37°C in a constant temperature incubator with 5% CO_2_ in DMEM medium (Gibco, USA), including 10% fetal bovine serum (HyClone, North America) and 100 *μ*g/ml penicillin and streptomycin.

### 2.3. CCK-8 Assay and Synergy Effect Analysis

143B cells were made into 1‐5 × 10^3^/l cell suspension and seeded in 96-well plates. Then, the cells were treated with ZA (0, 4, 8, 16, 32, 64, 128, and 256 *μ*M) or cisplatin (0, 0.4, 1.0, 2.5, 5.0, 10, 20, and 40 *μ*g/ml) alone or in combination. Finally, after CCK-8 reagents were added according to CCK-8 kits protocol and incubated for 0.5-4 hours in the cell culture incubator, the absorbance was measured at 450 nm in a microplate reader. After obtaining the data of cell activity, the synergy score was determined by the online application SynergyFinder 2.0 with four reference models with the “viability readout” [18]. And the inhibitory concentration 50 (IC_50_) value of cisplatin was calculated by the survival curve.

### 2.4. Colony Formation Assay

The cells were seeded in six-well plates at the appropriate density (500 cells/well) and allowed to culture for 24 h. Once the cells completely adhered, the cells were divided into six groups: control, cisplatin, ZA, ZA+cisplatin, ZA+cisplatin+NAC, and ZA+cisplatin+740 Y-P, which were treated with 32 *μ*M ZA, 5 *μ*g/ml cisplatin, 5 mM NAC, or 50 *μ*g/ml 740 Y-P for 48 h, respectively. Then, the cells were washed twice with PBS and cultured in a new medium. Eight days later, the cells were fixed with 4% paraformaldehyde and stained with crystal violet. The assay was repeated three times. The staining results were photographed, and the colonies with more than 50 cells were counted under an ordinary optical microscope.

### 2.5. Animals and Treatment

All experimental specific pathogen-free BALB/c nude mice (4-6 weeks old) (No. 2019-0012, license number: SCXK (Hubei), certification number: 42000800001149) were purchased from Hubei Provincial Center for Disease Control and Prevention (Hubei, China). All animal testing procedures comply with the Guidelines for the Care and Use of Laboratory Animals of the Chinese Animal Welfare Committee. The Animal Experimental Ethics Committee of Wuhan University Medical College approved this study (license number: 18026). All animals were kept under sterile standard environmental conditions, allowing free access to food and water. After one week of adaptive feeding, we injected 40 *μ*l 143B cells into the right tibial plateau of the nude mice with an insulin needle. When the tumor was visible, the mice were randomly assigned to one of four groups: control, cisplatin (2 mg/kg), and combined group (ZA: 2 mg/kg+cisplatin: 2 mg/kg). The above reagents were injected intraperitoneally 3 times once every 5 days. The weight and tumor size of mice were measured every 3 days. The tumor size was then calculated based on the following equation: tumor volume = (length × width^2^)/2. Finally, all nude mice were sacrificed with 2% isoflurane, the blood samples were collected, and the tumors and lower limbs were isolated for further analysis.

### 2.6. X-Ray and Micro-CT

Before the nude mice were sacrificed, the mice were anesthetized with 4% chloral hydrate and then examined tibia with X-rays under the mouse in vivo imager (including X-ray imaging module) (Bruker Xtreme BI, BRUKER, USA) and observed bone destruction. After the nude mice were sacrificed, the bones were soaked in ethanol. Then, we used a micro-CT scan (Quantum GX, PerkinElmer) to scan the tumor side tibia and femur and perform 3D reconstruction. Finally, we used the software (Analyze 12.0, PerkinElmer) to assess the trabecular bone microstructure with the parameters, including trabecular bone volume per tissue volume (BV/TV), trabecular number (Tb.N), trabecular thickness (Tb.Th), and trabecular separation (Tb.Sp) of the scanned femurs.

### 2.7. Immunofluorescence Staining and ROS Assay

After the cells were fixed with 4% formaldehyde for 15 min, the membranes were broken with 0.2% Triton X-100 for 5-10 min. Then, samples were incubated with 3% BSA at room temperature for 60 min. Soon afterward, they were incubated overnight at 4°C with primary antibodies in blocking buffer. After washing with TBST three times, the samples were incubated with 1 : 800 secondary antibody-labeled fluorescent for 60 min. The nucleus was then stained with DAPI for 5 min. After washing with PBS three times, fluorescence images were taken with confocal microscopy (Smartproof 5, Carl Zeiss, Oberkochen, Germany). 10 visual fields were collected for further analysis of each image. Differently, after the ROS reagent was directly added to the sample according to the protocol, it was immediately observed under a confocal microscope (Smartproof 5, Carl Zeiss, Oberkochen, Germany), and the ImageJ software (NIH, Bethesda, MD, USA) was used for quantitative analysis.

### 2.8. Formation of Osteoclast Cells and Bone Resorption Assay

Primary bone marrow cells were cultured in the complete medium supplemented with 50 ng/ml M-CSF for 3 days to obtain bone marrow-derived macrophages (BMMs). Then, we cultured the cells for 4 days with 50 ng/ml M-CSF and 50 ng/ml RANKL. To assess osteoclast survival, 32 *μ*M ZA and/or 5 *μ*g/ml cisplatin were added for the first two days. The samples were fixed with 4% paraformaldehyde and stained with the TRAP staining. As for bone resorption assay, after we cultured the BMMs in Osteo Assay Surface 24-well plates (Corning, Corning, NY, USA) for 24 h, a complete inducing medium replaced the previous medium. Similarly, 32 *μ*M ZA and/or 5 *μ*g/ml cisplatin were added for the first two days. The cells were rinsed with 10% sodium hypochlorite and water three times after 6 days. Finally, we used an ordinary light microscope to take pictures, and the ImageJ software was used for quantitative analysis.

### 2.9. TRAP Staining

After the cells were treated, TRAP staining of osteoclasts was performed according to the TRAP staining kit protocol. Briefly, after removing the culture medium, the cells were immobilized by adding 50 *μ*l of 10% paraformaldehyde. After washing with ddH_2_O, add tartrate-containing buffer and 50 *μ*l chromogenic substrate. After incubating at 37°C for 40 min, wash with ddH_2_O. Finally, we used Nikon NIS Elements BR light microscope (Nikon, Tokyo, Japan) for photograph. The staining intensity was analyzed by measuring the mean optical density (MOD) in 10 fields.

### 2.10. H&E, Ki-67 Staining, and Tunel Assay

The specimens were sectioned at 5 *μ*m for further morphological staining analysis. According to the protocol, H&E staining was operated as follows: after dewaxing in xylene for 10 min, the slices were immersed in 100%, 95%, 85%, and 70% ethanol for 3 min. Next, after dyeing with hematoxylin for 10 min, the color was separated with 70% ethanol. After washing with running water for 15 min, dye with 0.2% eosin for 3 min and then dehydrate by 70%, 85%, 95%, and 100% ethanol successively. Finally, xylene was used for transparency. Ki-67 and Tunel staining was followed by an immunohistochemical staining protocol described in this article [19]. All images were pictured with Nikon NIS Elements BR light microscope (Nikon, Tokyo, Japan) for further analysis.

### 2.11. RT-qPCR

The total RNA of cells and bone tissue was extracted by the method of TRIzol according to the manufacturer's guidelines. As for the quantitative real-time PCR (RT-qPCR), the reactions were acted in a 10 *μ*l system (1 *μ*l cDNA, reverse and forward primers, 3.6 *μ*l ddH2O, and 5 *μ*l SYBR Green Master Mix) with the Applied Biosystems PCR System (Thermo Fisher Scientific, USA): 95°C for 2 min, 40 cycles at 95°C for 15 s, and 60°C for 1 min. The cycle threshold (Ct) values were detected by the 2^−*ΔΔ*Ct^ method. And the relative mRNA expression was normalized with GAPDH. The primers of related genes are shown in Table S1.

### 2.12. Flow Cytometry

After different treatments, the experimental cells were centrifuged and collected after digestion with trypsin without EDTA. Then, we used an Annexin V-FITC Apoptosis Detection Kit (BB-4101-50T, Best Biotechnology, China) to detect apoptotic cells according to the protocol. In brief, after the cells were resuspended with 400 *μ*l 1x Annexin V binding buffer, 5 *μ*l Annexin V-FITC staining was added to the cell suspension, and the mixture was gently mixed and incubated at 2-8°C in the dark for 15 min. Then, PI dye solution was added and incubated at 2-8°C in the dark for 5 min. The prepared cells were immediately detected by CytoFLEX (Beckman Coulter, United States) flow cytometry. And the results were analyzed with the CyteExpert software (Beckman Coulter, United States). According to the instructions, the sum of UR and LR quadrant cells is the total apoptosis rate. The experiment was repeated three times for statistical analysis.

### 2.13. Western Blotting

The prepared cells were rinsed with PBS twice, and then, RIPA Lysis Buffer was used to lyse the cells for 30 min on ice to extract total protein, and the protein sample was then obtained when the supernatant was collected and transferred to the 1.5 ml precooled centrifuge tube after being centrifuged for 10 min at 12000g and 4°C. An equal amount of protein (100 *μ*l) was mixed with sodium dodecyl sulfate-polyacrylamide gel electrophoresis (SDS-PAGE) loading buffer (Beyotime, China) and heated at 95°C for 5 min. Finally, the protein sample obtained above was used for further protein quantification (p-PI3K, PI3K, p-AKT, AKT, MDR1, MRP1, and GAPDH). All results were repeated 3 times. The detailed steps of protein molecule separation and visualization were shown in our previous research [20]. The ImageJ software (NIH, Bethesda, MD, USA) was used to measure the gray values.

### 2.14. Statistical Analysis

SPSS 20 (SPSS Science Inc., Chicago, Illinois) and Prism 7 (GraphPad Software, La Jolla, CA, USA) were used for data statistics and graphing. Quantitative data were expressed as the mean ± SD. SNK-q was used for the comparison in two of multiple samples after one-way ANOVA that was used for the comparison of the control, single, and combined treatments. *P* < 0.05 was defined as statistically significant.

## 3. Results

### 3.1. Zoledronic Acid Enhanced Cisplatin-Induced Proliferation Inhibition and Apoptosis of Osteosarcoma Cells

**Figure 1:**
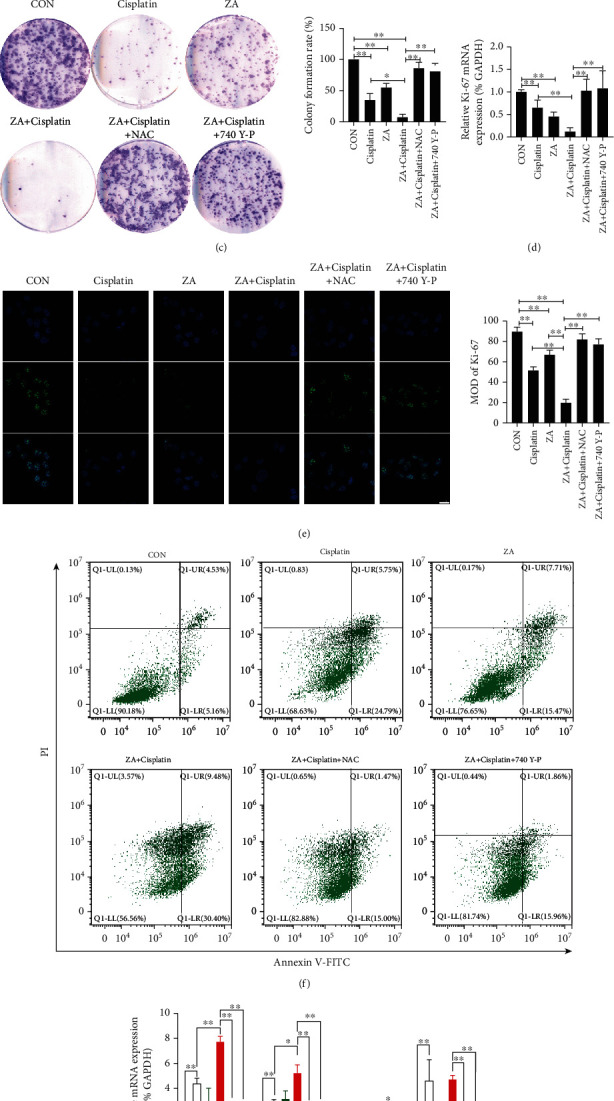
Zoledronic acid (ZA) increased cisplatin-induced apoptosis and proliferation inhibition in 143B cells. (a) Cell viability was measured by CCK-8 assay after 24 h in culture with the indicated concentrations of ZA/cisplatin/combination. (b) CCK-8 assay was performed to determine the cell viability of 143B cell after treatment with ZA/cisplatin/combination for 0, 24, 48, and 72 hours. (c) Cell clone formation of the 143B cells treated with cisplatin/ZA/ZA+cisplatin/ZA+cisplatin+NAC/ZA+cisplatin+740 Y-P. (d) The mRNA expression of Ki-67 was detected by RT-qPCR in 143B cells with the different treatment in the figures. (e) Immunofluorescence was used to detect Ki-67 protein expression in 143B cells treated by cisplatin/ZA/ZA+cisplatin/ZA+cisplatin+NAC/ZA+cisplatin+740 Y-P and quantification of the MOD value of the Ki-67 expression; scale bar = 25 *μ*m. (f) Flow cytometry was used to detect apoptosis induced by cisplatin/ZA/ZA+cisplatin/ZA+cisplatin+NAC/ZA+cisplatin+740 Y-P. (g) RT-qPCR was used to detect the mRNA expression of caspase 3/9, Bcl-2, and Bax. CON: control; ZA: zoledronic acid; NAC: N-acetyl-L-Cysteine; MOD: mean optical density; Ki-67: marker of proliferation Ki-67; Bcl-2: protein phosphatase 1, regulatory subunit 50; Bax: BCL2-associated X protein omega. The values were the means ± SD, *n* = 3; ^∗^*P* < 0.05, ^∗∗^*P* < 0.01.

Firstly, CCK-8 assay was used to detect the cell viability of 143B cells treated with ZA/cisplatin/combination in different concentrations and times. The results showed that the cell activity decreased in a concentration- or time-dependent manner whether in a separate group or the combined group, while the CCK-8 absorption curve in the combined group was significantly higher than alone or control (Figures 1(a) and 1(b); *P* < 0.05, *P* < 0.01). Cell clone and immunofluorescence were further used to measure the cell proliferation. The cell clone showed that clone formation was significantly lower in the combined group than either the cisplatin or ZA group, and all of that were lower than control (Figure 1(c); *P* < 0.01). The Ki-67 expression by RT-qPCR and immunofluorescence showed that Ki-67 mRNA and protein expressions in the cisplatin or ZA group were significantly lower than those in the control and higher than those in combination (Figures 1(d) and 1(e); *P* < 0.01). Furthermore, to investigate the apoptosis of 143B cells treated with ZA/cisplatin/combination, flow cytometry and RT-qPCR were used. The results showed that the expression of apoptosis-related genes (caspase 3/9 and Bax) and the apoptosis rate of 143B in the combined treatment group were significantly higher than those in the control and single treatment groups (Figures 1(f) and 1(g), S2; *P* < 0.05, *P* < 0.01), while the antiapoptosis gene (Bcl-2) expression was the lowest in the combined treatment. Furthermore, the synergy score of cisplatin and ZA was determined by SynergyFinder 2.0 [18]. The results showed that all the averaged synergy scores using four reference models were more than ten, indicating there is a good synergistic antiosteosarcoma effect between cisplatin and ZA (Figure S1a–d). In short, cisplatin in synergy with ZA could significantly induce the proliferation inhibition and apoptosis of 143B cells.

### 3.2. Oxidative Stress and PI3K/AKT Signaling Inhibition Mediated Cisplatin in Synergy with Zoledronic Acid Induced Apoptosis and Proliferation Inhibition of Osteosarcoma Cells

**Figure 2:**
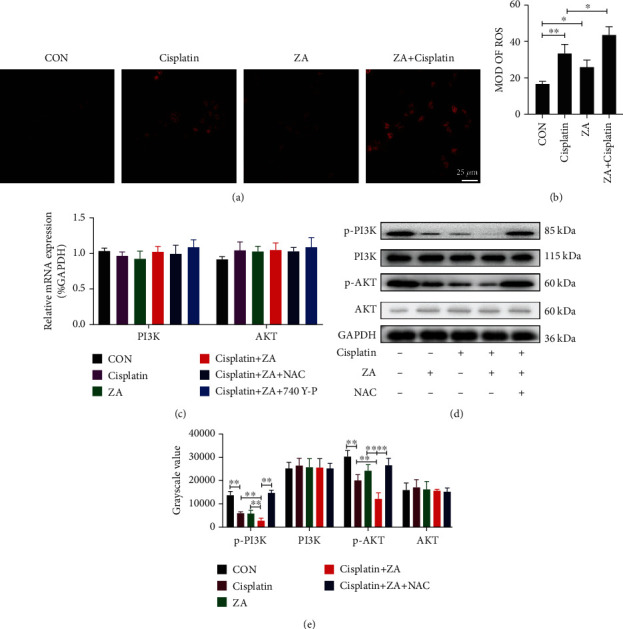
ROS-PI3K/AKT mediated the synergistic effect of ZA and cisplatin. (a) The ROS generated after ZA and cisplatin treated 143B cells alone or in combination, scale bar = 25 *μ*m. (b) The quantitative analysis of ROS immunofluorescence. (c) The mRNA expression of PI3K and AKT was detected by RT-qPCR in 143B cells with different treatment. (d) Western blotting was used to detect the phosphorylated protein expression of 143B PI3K and AKT after different treatments. (e) Grayscale analysis of p-PI3K, PI3K, p-AKT, and AKT protein expression. CON: control; ZA: zoledronic acid; MOD: mean optical density; p-PI3K: phospho-PI3 kinase; p-AKT: phospho-AKT; ROS: reactive oxygen species; NAC: N-acetyl-L-Cysteine. The values were the means ± SD, *n* = 3; ^∗^*P* < 0.05, ^∗∗^*P* < 0.01.

To further explore the mechanism of ZA and cisplatin enhancing antitumor, immunofluorescence was used to detect ROS generation after single or combined action of ZA and cisplatin. The results showed that the ROS generation after ZA or cisplatin treatment was significantly increased compared with the control, but the ROS of the ZA group was lower than that of the cisplatin group, while the ROS production after combined treatment was higher (Figures 2(a) and 2(b); *P* < 0.05, *P* < 0.01). The PI3K/AKT signaling is a crucial pathway related to the proliferation and apoptosis of osteosarcoma [21]. We further detected the expression of the PI3K/AKT pathway by RT-qPCR and Western blotting. The results showed that the phosphorylation of PI3K and AKT was significantly decreased in ZA or cisplatin alone than the control while there was no significant change in the mRNA and protein expression of PI3K and AKT, and the combined treatment was more significant than the single treatment group (Figures 2(c)–2(e); *P* < 0.05, *P* < 0.01). Furthermore, when we treated 143B cells with ROS scavenger N-acetyl-L-Cysteine (NAC) and 740 Y-P (an agonist for PI3K/AKT signaling pathway [22]) in combination with ZA+cisplatin, the results of phosphorylation inactivation of PI3K and AKT, clone formation and proliferation inhibition, and increased apoptosis were reversed (Figures 1(c)–1(g) and 2(d) and 2(e); *P* < 0.05, *P* < 0.01). Therefore, these results suggested that ZA+cisplatin could inhibit PI3K/AKT signaling activation by promoting ROS generation to induce apoptosis and proliferation inhibition of osteosarcoma cells.

### 3.3. Cisplatin in Synergy with Zoledronic Acid Inhibited Osteoclast Differentiation and Promoted the Apoptosis

**Figure 3:**
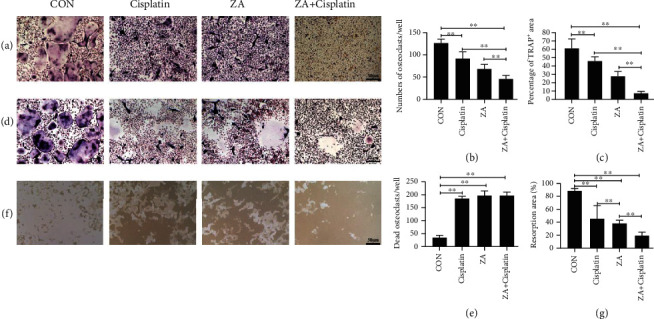
Cisplatin in synergy with zoledronic acid (ZA) inhibited osteoclast formation, survival, and activation. (a) The TRAP staining of BMMs treated with M-CSF and RANKL for 4 days in the presence of cisplatin/ZA+cisplatin. (b) The osteoclasts per well were counted. (c) The area of TRAP^+^ multinucleated cells (nuclei > 3) was quantified. (d) The mature osteoclasts were treated by cisplatin/ZA+cisplatin. (e) The dead osteoclasts per well were counted. (f) The bone resorption on Corning Osteo Assay 24-well plates of osteoclasts treated by cisplatin/ZA+cisplatin. (g) The resorption area was quantitative. CON: control; ZA: zoledronic acid. The values were the means ± SD, *n* = 3; scale bar = 50 *μ*m; ^∗^*P* < 0.05, ^∗∗^*P* < 0.01.

It has been reported that ZA can inhibit osteoclast differentiation and survival [23]. However, chemotherapy drugs such as cisplatin often caused complications such as bone loss [24]. Moreover, osteoclasts are the essential cells for osteosarcoma to invade and destroy the surrounding bone tissue [25]. Therefore, we examined whether ZA inhibited osteoclast differentiation and survival after cisplatin treatment. Physiologically, osteoclasts are derived from BMMs. Consequently, we treated the cells during the differentiation of BMMs into osteoclasts with cisplatin, ZA, or combination. The results showed that TRAP staining positive rate and the number of osteoclasts in the combined treatment group were significantly lower than those in the single treatment group, and both were lower than the control (Figures 3(a)–3(c); *P* < 0.05, *P* < 0.01). We further tested the effects of ZA and cisplatin on the survival of osteoclasts. Consistent with differentiation, the osteoclast apoptosis in the ZA+cisplatin group was more than that in the control, but it was not significantly different from the single treatment group (Figures 3(d) and 3(e); *P* < 0.05, *P* < 0.01). Moreover, the bone resorption of the combined treatment group was significantly higher than that of the single treatment group, and both were stronger than the control (Figures 3(f) and 3(g); *P* < 0.05, *P* < 0.01). The above results indicated that either ZA or cisplatin could inhibit osteoclast formation, survival, and activation, but the combination was more effective.

### 3.4. Zoledronic Acid Enhanced Cisplatin-Induced Tumor Inhibition In Vivo

**Figure 4:**
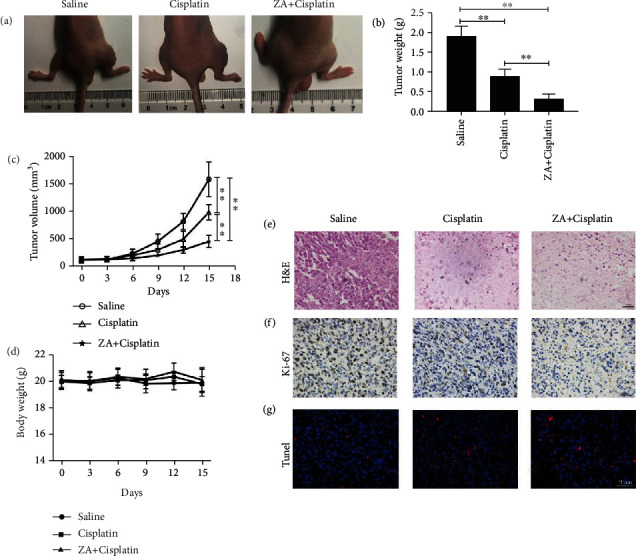
Cisplatin in synergy with zoledronic acid (ZA) inhibited osteosarcoma in vivo. (a) The tumor growth in nude mice. (b) Tumor weights were weighted after the mice were sacrificed. (c) Tumor volume was measured every 3 days. (d) Mouse body weights were measured per 3 days. (e) H&E staining of tumors, scale bar = 100 *μ*m. (f) Immunohistochemical analysis of Ki-67 protein expression in tumor tissues, scale bar = 100 *μ*m. (g) Tunel staining in tumor tissues indicated the apoptosis of tumor cells, scale bar = 50 *μ*m. H&E: hematoxylin-eosin staining; CON: control; ZA: zoledronic acid. The values were the means ± SD, *n* = 5; ^∗^*P* < 0.05, ^∗∗^*P* < 0.01.

To further explore the effects in vivo, we first established a nude mouse tibia orthotopic osteosarcoma model, and these model mice were then treated with cisplatin/ZA+cisplatin. The results showed that the tumors in the combined treatment group were significantly smaller visually than those in the single treatment and control (Figure 4(a)), and the weight of tumors was lower (Figure 4(b); *P* < 0.01). Furthermore, tumor volume and nude mouse body weight were measured, and tumor growth was significantly slower in the combined treatment group than in the other treatment and control (Figure 4(c); *P* < 0.05, *P* < 0.01), but there was no significant change in weight (Figure 4(d); *P* > 0.05). Further, we exfoliated the tumor and performed H&E, Ki-67, and Tunel staining. The results showed that the apoptosis and necrosis of tumor cells (Figures 4(e) and 4(g)) were significantly increased, while the proliferation (Figure 4(f)) was decreased. Finally, the function of the liver and kidney was tested because the hepatotoxicity and nephrotoxicity of chemotherapeutic drugs are the most common side effects [26, 27]. The results showed that the serum levels of ASL, ALT, CREA, and BUN in the combined group were not higher than those in the group treated by cisplatin, but they were all higher than those in the control (Figure S4a–d; *P* < 0.05, *P* < 0.01), indicating that the side effects of combined treatment were not higher than those of cisplatin alone.

### 3.5. Cisplatin in Synergy with Zoledronic Acid Attenuated Osteolytic Destruction

**Figure 5:**
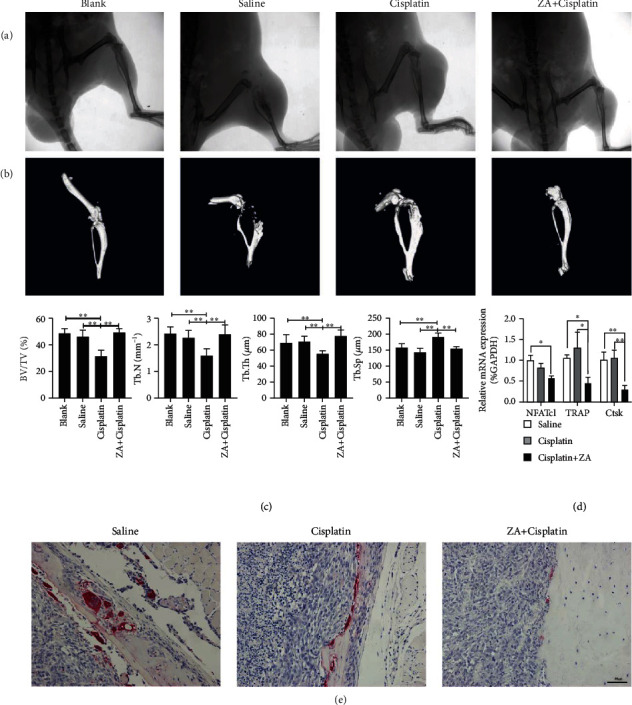
Cisplatin in synergy with zoledronic acid (ZA) attenuated osteolysis. (a) X-ray radiography of lower limb in the side of the tumor. (b) Three-dimensional reconstruction images of micro-CT. (c) Analysis of the femoral cancellous bone mass based on the data from micro-CT scan, including BV/TV, Tb.N, Tb.Th, and Tb.Sp. (d) The mRNA expression of NFATc1, TRAP, and Ctsk in the bone tissue at the site of tumor invasion. (e) The TRAP staining of the tibia containing the tumor, scale bar = 100 *μ*m. ZA: zoledronic acid; BV/TV: bone volume/total volume; Tb.N: trabecular number; Tb.Th: trabecular thickness; Tb.Sp: trabecular separation; NFATc1: nuclear factor of activated T cells 1; TRAP: triiodothyronine receptor auxiliary protein; Ctsk: cathepsin K. The values were the means ± SD, *n* = 3; ^∗^*P* < 0.05, ^∗∗^*P* < 0.01.

Biologically, osteoclasts and osteosarcoma cells interact to form a “vicious cycle” conducive to osteosarcoma growth and osteolysis [25]. Therefore, inhibition of osteolytic status is an important step in inhibiting the growth of osteosarcoma. In a nude mouse model of orthotopic osteosarcoma in the tibia, combined treatment with ZA and cisplatin resulted in significantly lower bone tissue destruction than that of the group treated with cisplatin alone or saline (Figure 5(a)). Three-dimensional reconstruction images of micro-CT further showed that there was less bone destruction in the combined treatment group (Figure 5(b)), and the decrease of femoral cancellous bone mass in the group treated with cisplatin could be reversed by the combined therapy, as manifested by a change in BV/TV, Tb.N, Tb.Th, and Tb.Sp (Figure 5(c); ^∗^*P* < 0.05, ^∗∗^*P* < 0.01). Finally, we detected the mRNA expression of marker genes of osteoclast differentiation (NFATc1) and activity (TRAP and Ctsk) and performed TRAP staining on the tumor side tibia tissue. The results showed that the marker genes of osteoclast differentiation and activity were lower significantly in ZA+cisplatin than those in the group treated with saline or cisplatin (Figure 5(d); ^∗^*P* < 0.05, ^∗∗^*P* < 0.01). And TRAP staining-positive cells were also significantly reduced, indicating that osteoclasts in bone tissue were inhibited after ZA+cisplatin treatment (Figure 5(e)). In short, ZA could inhibit the formation of osteoclasts between osteosarcoma and normal bone, breaking the “vicious circle” between it and tumor cells to inhibit osteolytic destruction.

## 4. Discussion

Osteosarcoma, with extremely high nausea, is originated from osteoblast and often occurs in long tubular bone [28, 29]. Studies have shown that about 20% of bone tumors are osteosarcoma, which is common in adolescents [30]. With the development of surgery and adjuvant chemotherapy, the long-term survival rate of patients with osteosarcoma is increasing. However, the 5-year survival rate is still less than 70%, and osteosarcoma is still the main cause of cancer death in children and adolescents [4, 31]. Chemotherapy is one of the most important methods in the treatment of osteosarcoma [32]. The commonly used chemotherapy drugs include cisplatin, MTX, ADM, IFO, vincristine (VCR), epirubicin (EPI), cyclophosphamide (CTX), and etoposide (VP-16), among which cisplatin, MTX, ADM, and IFO are the most common [33]. However, the application of chemotherapy drugs such as cisplatin can easily lead to drug resistance and a variety of adverse reactions such as bone loss, which are pivotal reasons for the bottleneck in the treatment of osteosarcoma [34]. Therefore, it is urgent to explore new strategies to enhance the efficacy of chemotherapy drugs and reduce the side effects.

Bisphosphonates are the first-line drugs for the treatment of osteoporosis, whereas ZA is the third generation of nitrogen-containing bisphosphonates [35, 36]. However, increasing studies have shown that ZA has the effect of not only treating osteoporosis but also antitumor. Liu et al. showed that ZA can block the interaction between breast cancer cells and regulatory T cells, thereby reducing breast cancer invasion [16]. Wang et al. also showed that ZA can regulate cell cycle and apoptosis to inhibit the growth of cervical cancer cells [17]. However, the understanding of ZA in osteosarcoma is still lacking, and its combined effect and mechanism with commonly used chemotherapy drugs are still unclear. In this study, cisplatin alone or in combination with ZA could reduce the activity of osteosarcoma cells, but the combined effect was more obvious. It took 48 hours for cisplatin alone to decrease the cell activity, but it decreased at 24 hours after the combination of cisplatin and ZA at the same dose. Moreover, after combined treatment whether in vitro or in vivo, the proliferation inhibition and apoptosis of osteosarcoma cells were more obvious, and the necrosis and growth inhibition of tumor were also obviously increased. The synergy score calculated by SynergyFinder was also more than ten. Therefore, these results showed that ZA could synergistically enhance the antitumor effect of cisplatin.

Cisplatin is one of the most commonly used and classic chemotherapy drugs in the treatment of osteosarcoma [37, 38]. However, its resistance limits its further application in clinic. Studies have shown that the mechanisms of cisplatin resistance include increased DNA repair, altered cellular aggregation, apoptosis resistance, and autophagy [39, 40]. Therefore, intervention in these mechanisms can inhibit the resistance of cisplatin. The PI3K/AKT signaling pathway is a classical tyrosine kinase cascade signal transduction pathway widely existing in organisms, which is involved in the occurrence and development of tumors, as well as the treatment and prognosis [41, 42]. PI3K is an intracellular phosphatidylinositol kinase with serine/threonine kinase activity. After phosphorylation of PI3K, AKT is further phosphorylated, and the PI3K/AKT pathway is activated, thus promoting the proliferation of tumor cells and inhibiting their apoptosis [43]. In this present study, the combination of ZA and cisplatin further inhibited the activation of the PI3K/AKT singling pathway in osteosarcoma cells than cisplatin alone. Further study showed that ZA and cisplatin combined treatment significantly increased the generation of ROS in osteosarcoma cells, while ROS scavengers could eliminate the inhibition of the PI3K/AKT pathway. What is more, in this study, ROS scavengers and the agonist of PI3K/AKT pathway (740 Y-P) could reverse the clone formation and proliferation inhibition and increased apoptosis of osteosarcoma induced by cisplatin+ZA. This was consistent with Deng's finding that dexamethasone can promote the generation of ROS in osteoblasts and thus inhibit the activation of PI3K/AKT [44]. However, how ROS induced the activation of the PI3K/AKT pathway remains to be further studied. Bugide et al. found that apoptosis resistance mediated by the PI3K/AKT pathway is closely related to cisplatin resistance [45]. Qiu et al. also proved that MNAT1 promotes cisplatin resistance of osteosarcoma cells via regulating the PI3K/AKT/mTOR pathway [46]. And another study by Zhao et al. further confirmed that activation of the PI3K/AKT signaling abolished poncirin-induced reduction of the mRNA and protein expression of multidrug resistance gene (MDR1, MPR1, and BCRP) in cisplatin-resistant osteosarcoma cells [47]. Therefore, we detected the effect of ZA+cisplatin on cisplatin resistance. The results showed that the IC_50_ values of cisplatin decreased significantly in the cisplatin-resistant osteosarcoma cells treated with 8 *μ*M ZA (this concentration had no effect on the cell viability) (Figure S3a; ^∗^*P* < 0.05, ^∗∗^*P* < 0.01). And the apoptosis rate increased, and the mRNA and protein expression of the multidrug resistance gene (MDR1, MPR1) decreased in the cisplatin-resistant osteosarcoma cells treated with cisplatin+ZA (Figure S3b–e; ^∗^*P* < 0.05, ^∗∗^*P* < 0.01). In conclusion, the combination of ZA and cisplatin can inhibit ROS-PI3K/AKT signaling to enhance the antitumor efficacy and drug resistance of cisplatin.

Physiologically, osteoclasts remove old bone and osteoblasts form new bone, maintaining bone homeostasis. The formation and activation of osteoclasts require two factors: RANKL and macrophage colony-stimulating factor [48]. It has been found that osteosarcoma cells can produce RANKL, which can promote the production of osteoclasts and osteolysis [25], and activated osteoclasts can secrete a variety of cytokines, such as TGF*β*, IGFs, and FGFs [9–11], to further promote the growth of tumor cells and form a “vicious circle.” Therefore, inhibiting the formation and activation of osteoclasts is also the key to suppress the growth of osteosarcoma. In this study, we found that ZA and cisplatin could significantly inhibit osteoclast differentiation, promote osteoclast death, and inhibit bone resorption in vitro. In vivo, we also found that in the combined treatment group, the number of osteoclasts at the junction of tumor and bone decreased significantly, and the destruction of bone by tumor decreased significantly. Interestingly, our study and previous studies have shown that cisplatin can reduce bone mass [24], but we also found that cisplatin could inhibit osteoclast formation, survival, and activity. Osteoblasts are another important factor in maintaining bone mass [49]. Therefore, the decrease in bone mass induced by cisplatin might be related to the inhibition of osteoblasts. In addition, it has been found that there is an important relationship between the PI3K/AKT signal pathway and osteoclast differentiation, survival, and the interaction of many kinds of cytokines [50, 51]. However, this study found that ZA and cisplatin could inhibit the activation of the PI3K/AKT signaling pathway in osteosarcoma cells, which might also be the reason for inhibiting the survival and formation of osteoclasts and reducing bone destruction, but it needs further confirmation.

## 5. Conclusion

In conclusion, this study confirmed that ZA can enhance the antiosteosarcoma effect of cisplatin and ameliorate chemoresistance in vivo and in vitro. The mechanism is related to the increased generation of ROS and inhibition of PI3K/AKT signal pathway activation. Moreover, the combination of ZA and cisplatin could inhibit osteoclast differentiation, survival, and activation, thus breaking the “vicious circle” to inhibit tumor growth and osteolysis destruction. Therefore, ZA may be used as an adjuvant chemotherapy drug beneficial to the treatment of osteosarcoma.

## Data Availability

The data that support the findings of this study are available from the corresponding author upon reasonable request.

## Supplementary Materials

Supplementary Materials Table S1: the primers used for a real-time quantitative polymerase chain reaction. *GAPDH*_(1)_: glyceraldehyde 3-phosphate dehydrogenase from human; Ki-67: marker of proliferation Ki-67; caspase 3: apoptosis-related cysteine protease 3; caspase 9: apoptosis-related cysteine protease 9; Bcl-2: protein phosphatase 1, regulatory subunit 50; Bax: BCL2-associated X protein omega; PI3K: phosphatidylinositol-4,5-bisphosphate 3-kinase; AKT: AKT serine/threonine kinase 1; MDR1: ATP binding cassette subfamily B member 1; MRP1: ATP binding cassette subfamily C member 1; *GAPDH*_(2)_: glyceraldehyde 3-phosphate dehydrogenase from mouse; NFATc1: nuclear factor of activated T cells 1; TRAP: triiodothyronine receptor auxiliary protein; Ctsk: cathepsin K. Figure S1: the combination of cisplatin and zoledronic acid (ZA) showed a synergistic effect against the osteosarcoma cell. The synergistic effect was analyzed with SynergyFinder by four reference models. The synergy delta score was calculated by HAS (a), Loewe (b), Bliss (c), and ZIP (d) reference models, respectively. The values in the center of each panel showed the highest synergy score. HAS, Loewe, Bliss, and ZIP Synergy scores > 0 indicate synergism (red regions), while the scores < 0 suggest antagonism (green regions). Figure S2: detection of apoptosis rate of 143B cells by flow cytometry. 143B cell was treated with cisplatin (5 *μ*g/ml) and/or ZA (32 *μ*M) or cisplatin+ZA+NAC or cisplatin+ZA+740 Y-P. CON: control; ZA: zoledronic acid; NAC: N-acetyl-L-Cysteine. The values were the means ± SD, *n* = 3; ^∗^*P* < 0.05, ^∗∗^*P* < 0.01. Figure S3: ZA attenuated the resistance of osteosarcoma cells to cisplatin. (a) After 143B/CDDP was treated with 8 *μ*M ZA for 12 h, the IC_50_ value of cisplatin was analyzed with a CCK-8 kit. (b, c) Flow cytometry was used to detect apoptosis of 143B/CDDP cells induced by cisplatin (5 *μ*g/ml)/ZA (32 *μ*M)/ZA+cisplatin. (d) RT-qPCR was used to detect the mRNA expression of MDR1 and MRP1 in 143B/CDDP cells treated with 8 *μ*M ZA. (e) The protein expression of MDR1 and MRP1 was analyzed by Western blotting. CON: control; ZA: zoledronic acid; MDR1: ATP binding cassette subfamily B member 1; MRP1: ATP binding cassette subfamily C member 1. The values were the means ± SD, *n* = 3; ^∗^*P* < 0.05, ^∗∗^*P* < 0.01. Figure S4: the hepatotoxicity and nephrotoxicity in the nude mice bearing 143B osteosarcoma cell treated with cisplatin or/and zoledronic acid. The serum concentration of ASL (a), ALT (b), CREA (c), and BUN (d) detected by an automatic biochemical analyzer. ZA: zoledronic acid; AST: aspartate aminotransferase; ALT: alanine aminotransferase; BUN: blood urine nitrogen; CREA: creatinine. The values were the means ± SD, *n* = 5; ^∗^*P* < 0.05, ^∗∗^*P* < 0.01.
