# Supplementary material for: Zoledronic Acid Enhanced the Antitumor Effect of Cisplatin on Orthotopic Osteosarcoma by ROS-PI3K/AKT Signaling and Attenuated Osteolysis
Source: Oxid Med Cell Longev. 2021 Mar 30;2021:6661534. doi: 10.1155/2021/6661534 (PMC8026287; doi:10.1155/2021/6661534)
Supplement: Supplementary Materials — Table S1: the primers used for a real-time quantitative polymerase chain reaction. GAPDH(1): glyceraldehyde 3-phosphate dehydrogenase from human; Ki-67: marker of proliferation Ki-67; caspase 3: apoptosis-related cysteine protease 3; caspase 9: apoptosis-related cysteine protease 9; Bcl-2: protein phosphatase 1, regulatory subunit 50; Bax: BCL2-associated X protein omega; PI3K: phosphatidylinositol-4,5-bisphosphate 3-kinase; AKT: AKT serine/threonine kinase 1; MDR1: ATP binding cassette subfamily B member 1; MRP1: ATP binding cassette subfamily C member 1; GAPDH(2): glyceraldehyde 3-phosphate dehydrogenase from mouse; NFATc1: nuclear factor of activated T cells 1; TRAP: triiodothyronine receptor auxiliary protein; Ctsk: cathepsin K. Figure S1: the combination of cisplatin and zoledronic acid (ZA) showed a synergistic effect against the osteosarcoma cell. The synergistic effect was analyzed with SynergyFinder by four reference models. The synergy delta score was calculated by HAS (a), Loewe (b), Bliss (c), and ZIP (d) reference models, respectively. The values in the center of each panel showed the highest synergy score. HAS, Loewe, Bliss, and ZIP Synergy scores > 0 indicate synergism (red regions), while the scores < 0 suggest antagonism (green regions). Figure S2: detection of apoptosis rate of 143B cells by flow cytometry. 143B cell was treated with cisplatin (5 μg/ml) and/or ZA (32 μM) or cisplatin+ZA+NAC or cisplatin+ZA+740 Y-P. CON: control; ZA: zoledronic acid; NAC: N-acetyl-L-Cysteine. The values were the means ± SD, n = 3; ∗P < 0.05, ∗∗P < 0.01. Figure S3: ZA attenuated the resistance of osteosarcoma cells to cisplatin. (a) After 143B/CDDP was treated with 8 μM ZA for 12 h, the IC50 value of cisplatin was analyzed with a CCK-8 kit. (b, c) Flow cytometry was used to detect apoptosis of 143B/CDDP cells induced by cisplatin (5 μg/ml)/ZA (32 μM)/ZA+cisplatin. (d) RT-qPCR was used to detect the mRNA expression of MDR1 and MRP1 in 143B/CDDP cells treated with 8 μM [file 6661534.f1.doc]

**Table S1．The primers used for a real-time quantitative polymerase chain reaction.**

| **Genes** | **Forward Primer** | **Reverse Primer** | **Annealing(℃)** |
| --- | --- | --- | --- |
| *GAPDH_（1）_* | GATTCCACCCATGGCAAATTC | GTCATGAGTCCTTCCACGATAC | 59 |
| *Ki-67* | CCAGCAGTAGGTGAAGAGAAAG | TCTCCATCCCAGTTCCATAGT | 59 |
| *Caspase 3* | AATGGACCTGTTGACCTGAAA | CCAGGAGCCATCCTTTGAAT | 61 |
| *Caspase 9* | CTAACAGGCAAGCAGCAAAG | GGAAAGTAGAGTAGGACACAAAGA | 58 |
| *Bcl-2* | TGGGAGAACAGGGTACGATAA | GAGACAGCCAGGAGAAATCAAA | 59 |
| *Bax* | GATGATTGCCGCCGTGGA | AGAGGAGGCCGTCCCAA | 62 |
| *PI3K* | CGTGCATGTGGGATGTATTTG | CAAGCCTGAGGTTTCCTAGTT | 59 |
| *AKT* | GTGATCCTGGTGAAGGAGAAG | CACAATCTCAGCGCCATAGA | 60 |
| *MDR1* | GAGTGGGCACAAACCAGATA | CTGACTCACCACACCAATGA | 60 |
| *MRP1* | CAAGTCATCCTTGCTCTCTACC | CAGACTTCTTCAGCACCTTCA | 62 |
| *GAPDH_（2）_* | GGAGAAACCTGCCAAGTATGA | CCTGTTGCTGTAGCCGTATT | 60 |
| *NFATc1* | AGTCCAACTACTCCTACCCATAC | CTGTAGCGTGAGAGGTTCATTC | 62 |
| *TRAP* | CTGCTGGGCCTACAAATCATA | GGGAGTCCTCAGATCCATAGT | 58 |
| *Ctsk* | CAGTGGGAGCTATGGAAGAAG | TTGGAAGGCAGTGGTCATATAG | 59 |

*GAPDH_（1）_*, glyceraldehyde 3-phosphate dehydrogenase from human; Ki-67, marker of proliferation Ki-67; Caspase 3, apoptosis-related cysteine protease 3; caspase 9, apoptosis-related cysteine protease 9; Bcl-2, protein phosphatase 1, regulatory subunit 50; Bax, BCL2-associated X protein omega; PI3K, phosphatidylinositol-4,5-bisphosphate 3-kinase; AKT, AKT serine/threonine kinase 1; MDR1, ATP binding cassette subfamily B member 1; MRP1, ATP binding cassette subfamily C member 1; *GAPDH_(2）_*glyceraldehyde 3-phosphate dehydrogenase from mouse; NFATc1, nuclear factor of activated T cells 1; TRAP, triiodothyronine receptor auxiliary protein; Ctsk, cathepsin K.


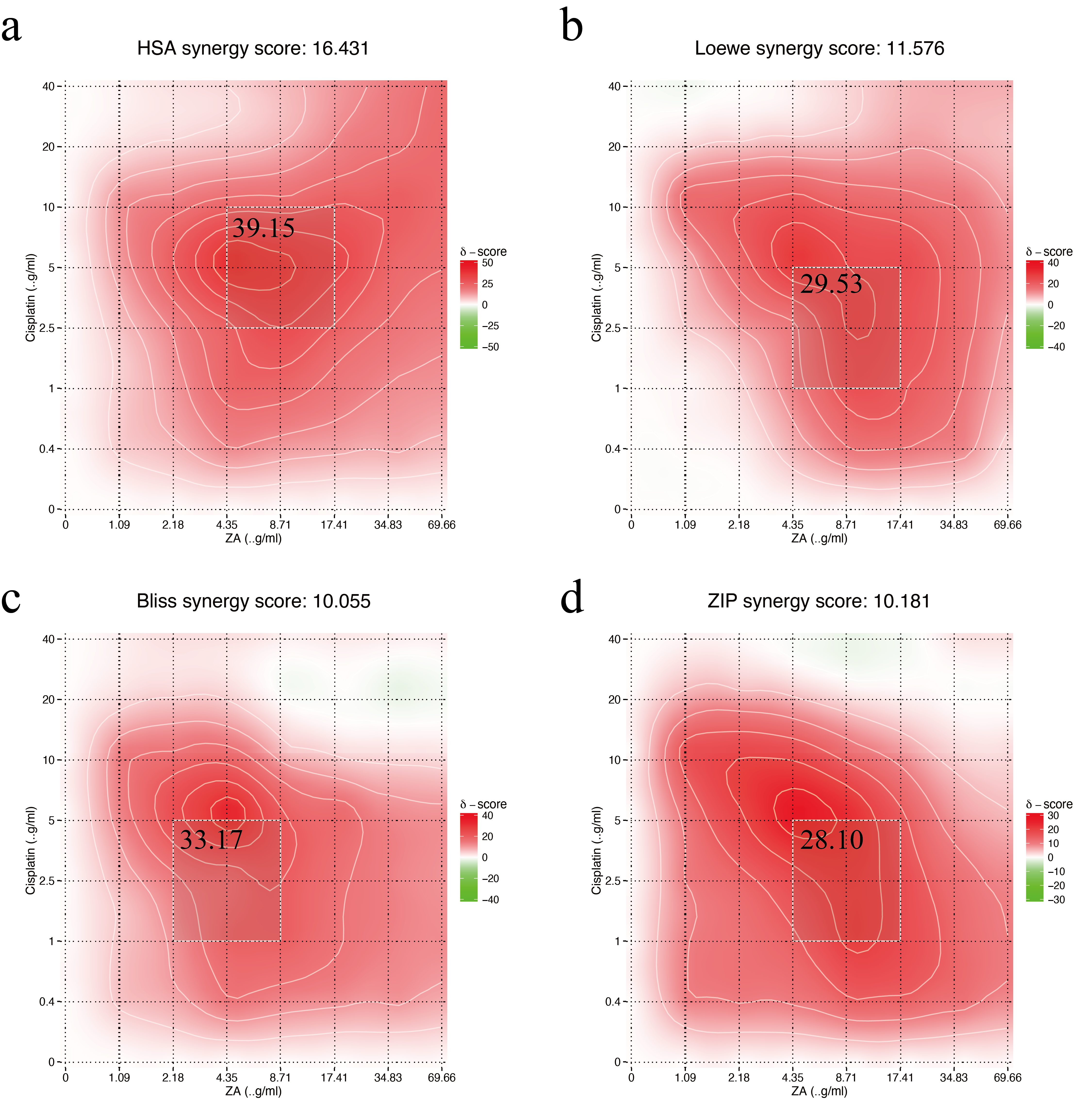


**Figure S1. The combination of cisplatin and zoledronic acid (ZA) showed a synergistic effect against the osteosarcoma cell.** The synergistic effect was analyzed with SynergyFinder by four reference models. The synergy delta score was calculated by HAS (a), Loewe (b), Bliss (c) and ZIP (d) reference models, respectively. The values in the center of each panel showed the highest synergy score. HAS, Loewe, Bliss and ZIP Synergy scores > 0 indicate synergism (red regions), while the scores < 0 suggest antagonism (green regions).

**Figure S2. Detection of apoptosis rate of 143b cells by flow cytometry.** 143b cell was treated with cisplatin (5 μg/ml) and/or ZA (32 μM), or Cisplatin+ZA+NAC or Cisplatin+ZA+740 Y-P. CON, control; ZA, zoledronic acid; NAC, N-acetyl-L-Cysteine; The values were the means ± SD, n=3; **P*<0.05, ***P*<0.01.


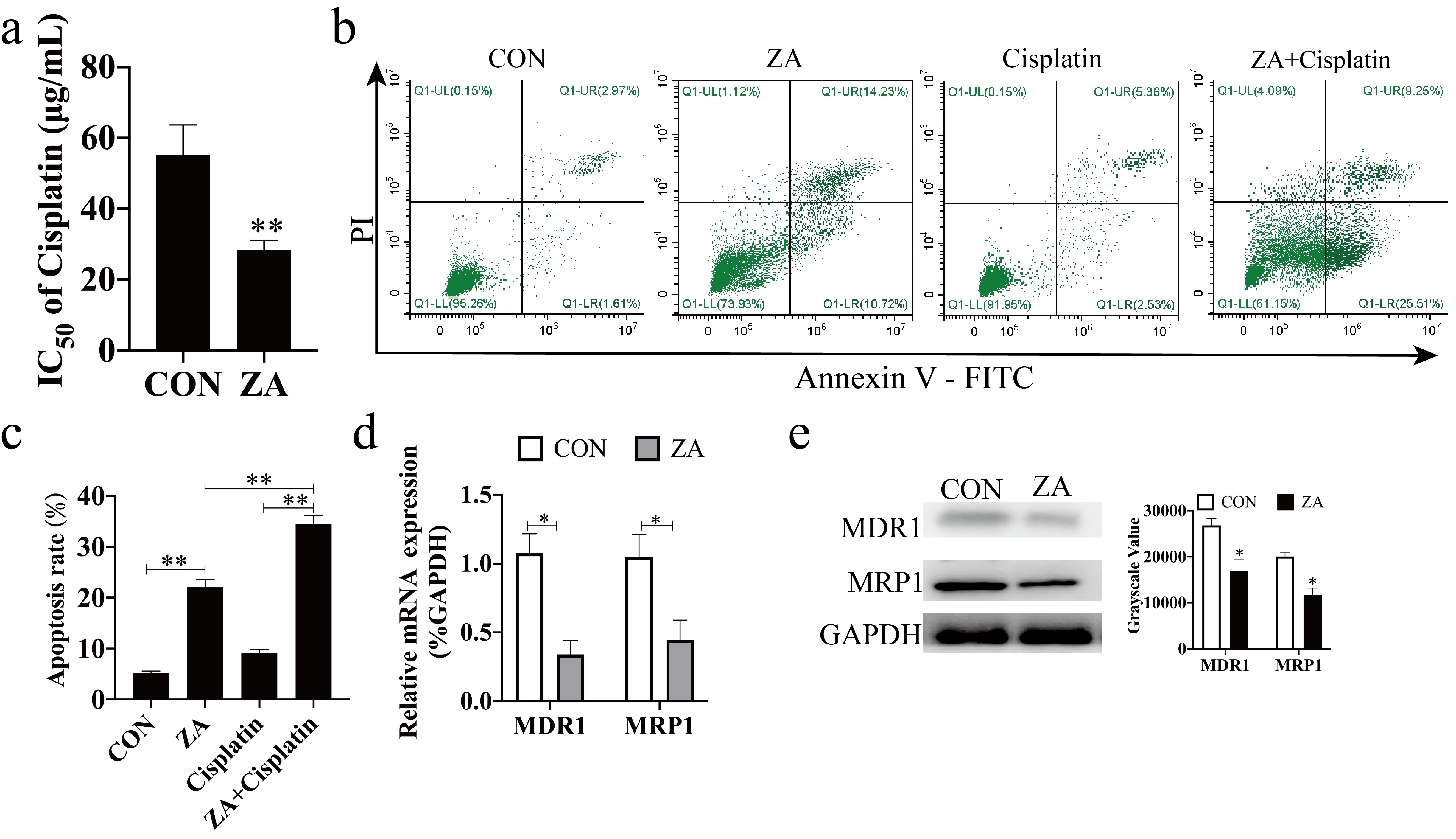


**Figure S3. ZA attenuated the resistance of osteosarcoma cells to cisplatin.** (a)After 143b/CDDP was treated with 8 μM ZA for 12h, the IC_50_ value of cisplatin was analyzed with a CCK-8 kit. (b, c) Flow cytometry was used to detect apoptosis of 143b/CDDP cells induced by cisplatin (5 μg/ml) /ZA (32 μM) /ZA+cisplatin. (d) RT-qPCR was used to detect the mRNA expression of MDR1 and MRP1 in 143b/CDDP cells treated with 8 μM ZA. (e) The protein expression of MDR1 and MRP1 were analyzed by Western blotting. CON, control; ZA, zoledronic acid; MDR1, ATP binding cassette subfamily B member 1; MRP1, ATP binding cassette subfamily C member 1. The values were the means ± SD, n=3; **P*<0.05, ***P*<0.01.


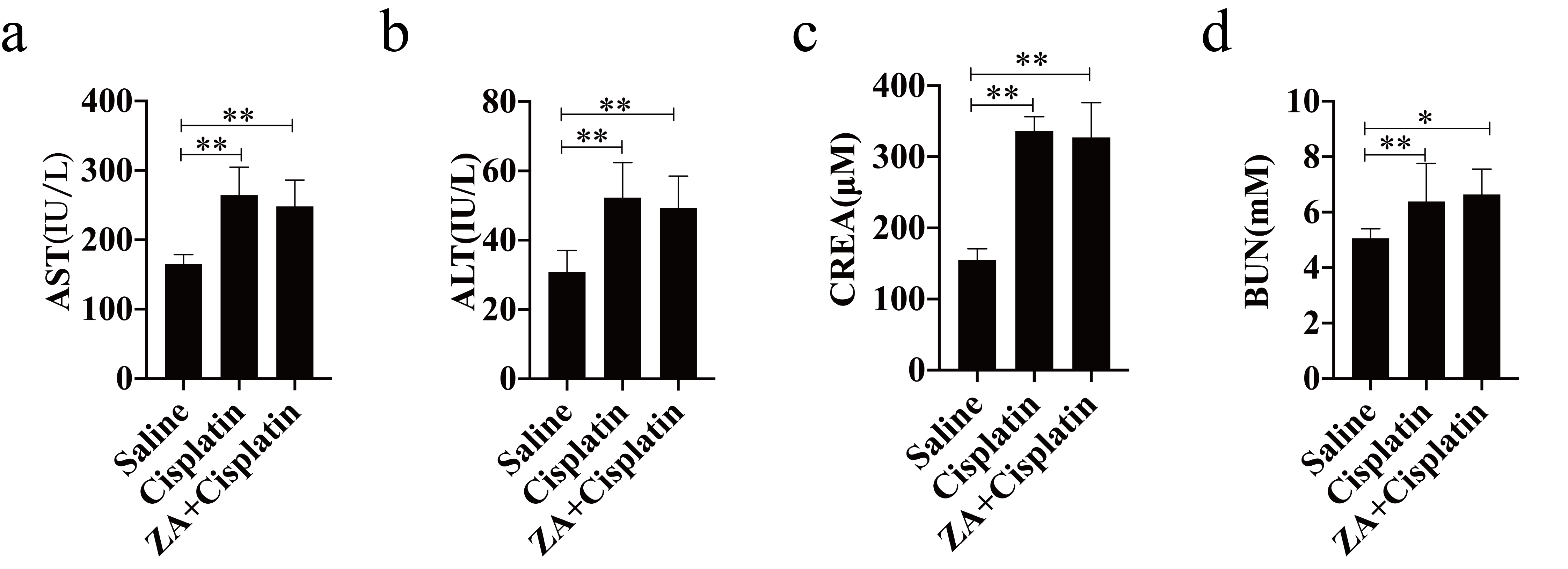


**Figure S4. The hepatotoxicity and nephrotoxicity in the nude mice bearing 143b osteosarcoma cell treated with cisplatin or/and zoledronic acid.** The serum concentration of ASL (a), ALT (b), CREA (c) and BUN (d) detected by an automatic biochemical analyzer. ZA, zoledronic acid; AST, aspartate aminotransferase; ALT, alanine aminotransferase; BUN, blood urine nitrogen; CREA, creatinine. The values were the means ± SD, n=5; ^*^*P*<0.05, ^**^*P*<0.01.
